# New interfaces on MiD51 for Drp1 recruitment and regulation

**DOI:** 10.1371/journal.pone.0211459

**Published:** 2019-01-31

**Authors:** Jun Ma, Yujia Zhai, Ming Chen, Kai Zhang, Quan Chen, Xiaoyun Pang, Fei Sun

**Affiliations:** 1 National Laboratory of Biomacromolecules, CAS Center for Excellence in Biomacromolecules, Institute of Biophysics, Chinese Academy of Sciences, Beijing, China; 2 University of Chinese Academy of Sciences, Beijing, China; 3 State Key Laboratory of Biomembrane and Membrane Biotechnology, Institute of Zoology, Chinese Academy of Sciences, Beijing, China; Chinese University of Hong Kong, CHINA

## Abstract

Mitochondrial fission is facilitated by dynamin-related protein Drp1 and a variety of its receptors. However, the molecular mechanism of how Drp1 is recruited to the mitochondrial surface by receptors MiD49 and MiD51 remains elusive. Here, we showed that the interaction between Drp1 and MiD51 is regulated by GTP binding and depends on the polymerization of Drp1. We identified two regions on MiD51 that directly bind to Drp1, and found that dimerization of MiD51, relevant to residue C452, is required for mitochondrial dynamics regulation. Our Results have suggested a multi-faceted regulatory mechanism for the interaction between Drp1 and MiD51 that illustrates the potentially complicated and tight regulation of mitochondrial fission.

## Introduction

Mitochondria are highly dynamic organelles that constantly undergo fusion, fission and move along the cytoskeleton [1]. Beyond the primary function of mitochondrial dynamics in controlling organelle shape, size, number and distribution, it is clear that dynamics are also crucial to specific physiological functions, such as cell cycle progression, quality control and apoptosis [2–5]. Dysfunction in mitochondrial dynamics has been implicated a variety of human diseases, including neurodegenerative diseases, the metabolism disorder diabetes and cardiovascular diseases [6,7].

Mitochondrial fission is mediated by multi-factors, such as dynamin-related protein Drp1 (Dnm1p in yeast) and its receptors on mitochondrial outer membrane, dynamin-2 (Dyn2) and endoplasmic reticulum [8,9]. However, Drp1 protein is mostly localized in the cytoplasm and must be recruited to the mitochondria by receptors on the mitochondrial outer membrane in response to specific cellular cues [10]. After targeting, Drp1 self-assembles into large spirals in a GTP-dependent manner and then contributes to mitochondrial membrane fission via GTP hydrolysis [5,11]. In yeast, the integral outer membrane protein fission protein 1 (Fis1) interacts with two adaptor proteins, Caf4 and Mdv1, providing an anchoring site for Dnm1p recruitment. In mammals, three integral outer membrane proteins, Mff, MiD51 and MiD49, were identified as receptors recruiting Drp1 to mitochondria. Overexpression of Mff induces Drp1 recruitment and mitochondrial fission [12–14]. MiD51 and MiD49 are anchored in the mitochondrial outer membrane via their N-terminal ends, and most of the protein is exposed to the cytosol. MiD51 and MiD49 specifically interact with and recruit Drp1 to mitochondria and then facilitate Drp1-directed mitochondrial fission [15]. It is notable that the expression of both MiD49 and MiD51 appears to be up-regulated in pulmonary arterial hypertension (PAH), one characteristic of which is rapid cell division associated with Drp1-mediated mitochondrial division [16]. And knock-down of endogenously elevated levels of MiD49 or MiD51 induces mitochondrial fusion [16].

Crystal structures of the cytosolic domains of MiD49 and MiD51 were reported and indicate that these proteins possess nucleotidyltransferase (NTase) folds and belong to the NTase family [17,18]. However, both proteins lack the catalytic residues required for transferase activity [17,18]. MiD51 does bind adenosine diphosphate (ADP) as a cofactor, but MiD49 lacks this capacity. The recruitment of Drp1 to the mitochondrial outer membrane by MiD51 was also addressed by two studies [17,18] where a single exposed loop corresponding to residues 238–242 on the surface of MiD51 was identified as the Drp1-binding loop. Mutants lacking this active loop are defective in recruiting Drp1 to the mitochondrial surface. But there are still paradoxical and unclear aspects about the molecular mechanisms of Drp1 recruitment[17,18]. In addition, Losón *et al* [18]proposed that MiD51 forms a dimer mainly via electrostatic interactions within the N-terminal segments and that dimerization is required for MiD51 mitochondrial fission activity but not Drp1 recruitment. Dimerization of MiD51 was not even addressed in Richter *et al*’s work[17]. Moreover, it is still not clear how the fission activity of MiD51 is co-regulated with Drp1.

Here, by combining structural biology, biochemical and biophysical techniques, we reveal that the interaction between MiD51 and Drp1 is regulated by the nucleotide binding state and polymerization of Drp1, and identify a second region on MiD51 that is important for Drp1 binding. We also show that MiD51 can form a homodimer through an interface close to residue C452 and is required for the regulation of mitochondrial dynamics. These results provide further insight into the molecular mechanism of interaction between Drp1 and MiD51, which plays key roles in mitochondrial fission regulation.

## Results

### Interaction between MiD51 and Drp1 is dependent on the oligomerization of Drp1

**Fig 1:**
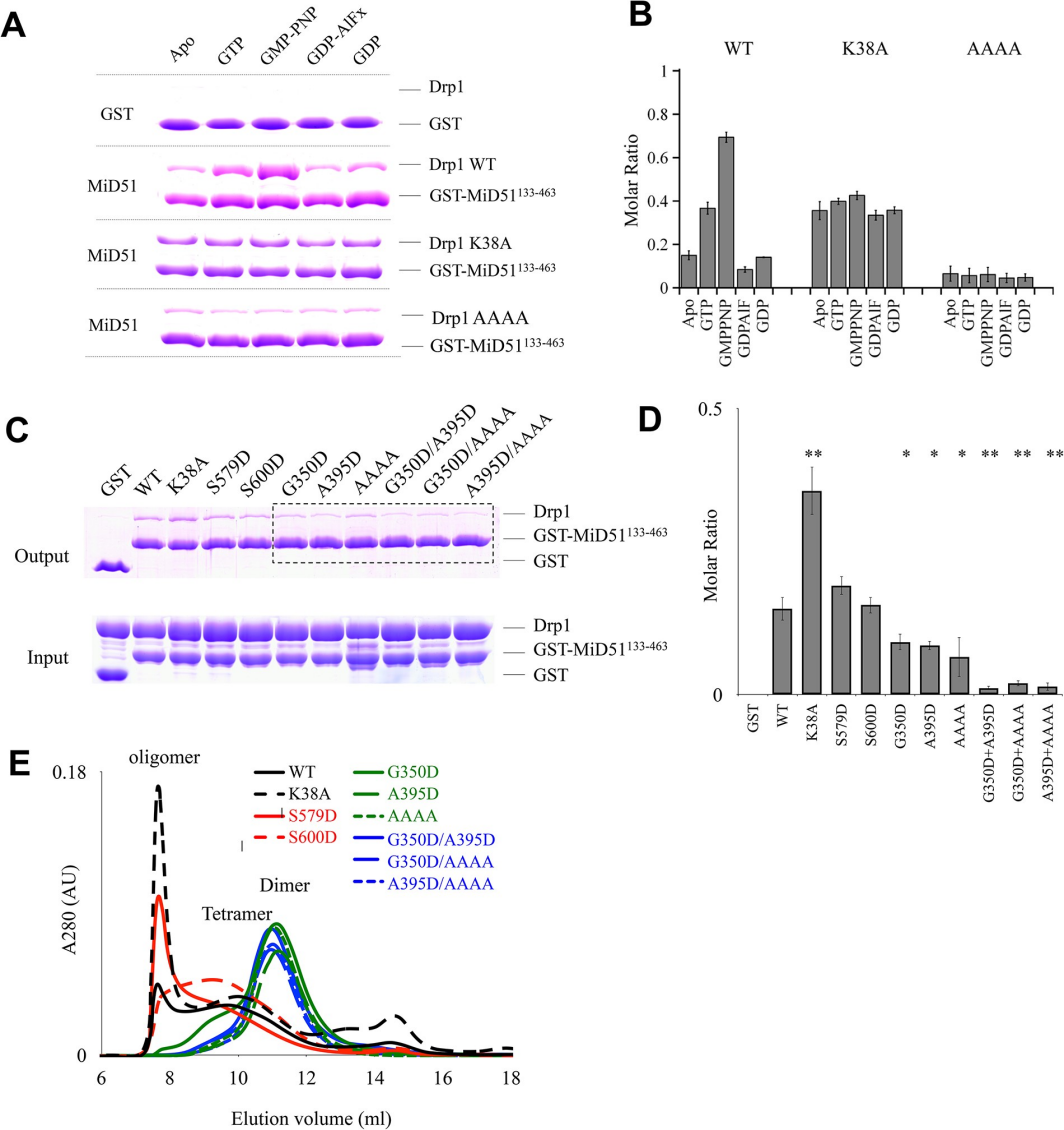
The cytoplasmic domain of MiD51 interacts with Drp1 is dependent on Drp1 oligomerization. (A) Pull-down assays were performed to test the binding of purified Drp1 or mutants to GST-MiD51^133-463^ in the presence of different nucleotides. MiD51 and Drp1 or their mutants were mixed evenly before adding to the same amount of resin with the same volume to ensure equal amount of protein was used, and then 1 mM nucleotide at final concentration was added. (B) Quantification of the results in (A). (C) Pull-down assays were performed to demonstrate that the binding of Drp1 to MiD51 depends on Drp1 oligomerization. Purified GST, and GST-MiD51^133-463^ were loaded onto Glutathione Sepharose beads, and incubated with wild-type and mutated Drp1 to test their binding by SDS-PAGE. (D) Quantification of the results in (C). The binding affinity is expressed as molar ratio of Drp1 to MiD51 mutants. Data are shown as mean ± SEM of three independent experiments performed in triplicate, * P < 0.05; ** P < 0.005 compared to wild-type. (E) Size-exclusion chromatography profiles of Drp1 and Drp1 mutants as indicated. Size-exclusion chromatography was performed with the size-exclusion column Superdex 200 PC 3.2/20 (GE Healthcare). The elution peak at ~11 ml represents the Drp1 dimer.

It was reported that the N-terminus of MiD51 is anchored in the mitochondrial outer membrane and the C-terminus is cytosolic (S1A Fig). The cytosolic domain MiD51^133-463^ can fold into a compact structure and are required for binding to Drp1[17,18]. Considering that MiD51 does not undergo a conformational change upon ADP binding and mutants defective in ADP binding are still capable of recruiting Drp1[18], we speculate that the roles of ADP or its analogues in regulating MiD51 are independent of Drp1 binding. But Drp1 is a GTPase protein belonging to the Dynamin super-family, and it can bind and hydrolyze GTP. We speculated whether MiD51 could selectively and dynamically recruit Drp1 under different nucleotide states. To test this, we performed GST pull-down assays in the presence of GTP, GDP, GDP+AlF_X_ and non-hydrolyzed GTP analogs GMP-PNP. We found that MiD51^133-463^ interacts with the GMP-PNP-bound state of Drp1 with high affinity, and this affinity is even higher than for the GTP bound state (Fig 1A and 1B, S3A Fig). Under GDP+AlF_X_ and GDP conditions, the strength of the interaction between MiD51^133-463^ and Drp1 is almost the same as for the apo state (Fig 1A and 1B, S3A Fig). However, the K38A mutant of Drp1, which is defective in nucleotide hydrolysis [19], appears to have no difference in binding affinity to MiD51^133-463^ under different nucleotide states (Fig 1A and 1B, S3A Fig). Based on these results we conclude that the interaction between MiD51 and Drp1 undergoes changes during the process of GTP hydrolysis.

It is well established that Drp1 either in the presence of GTP or GMP-PNP forms oligomeric assemblies[19,20]. To further clarify that whether the apparent greater binding between MiD51 and Drp1 in the presence of GTP or GMP-PNP relies on the oligomerization of Drp1, we made a series of Drp1 mutations and tested their effects on binding to MiD51. In previous studies [21–25], Drp1 residues G350, G363, R376, A395 and G401 play important roles in its polymerization. We designed a series of mutants where these residues were changed to Asp and monitored their ability to polymerize using size-exclusion chromatography. We found that G350D and A395D behaved differently from wild-type Drp1 in size-exclusion chromatography (Fig 1E), suggesting defective oligomerization. Similar defects in oligomerization were also observed for the AAAA mutant form of Drp1 (^401^GPRP^404^→AAAA), [23]. In addition, the compound mutants G350D/A395D, G350D/AAAA and A395D/AAAA showed a severe reduction in oligomerization. Our results indicate that the A395, G350 and GPRP (401–404) residues are involved in the polymerization of Drp1, consistent with previous studies[23]. We next assessed the interactions between MiD51^133-463^ and Drp1 mutants. We found that compared to wild type, Drp1 oligomerization mutants G350D, A395D, AAAA, G350D/A395D, G350D/AAAA and A395D/AAAA have reduced affinity for MiD51^133-463^ (Fig 1C), confirmed by quantification (Fig 1D), and oligomerization mutant Drp1-AAAA generally has the same binding affinity for MiD51^133-463^ in the presence of different nucleotides (Fig 1A and 1B). We also designed other Drp1 mutant proteins, targeting residues not responsible for oligomerization, such as dominant-negative GTPase mutation K38A, and phosphorylation site mutants S579D and S600D (related to S616 and S637 in Drp1 isoform 1). We found that these three mutant proteins behaved similarly to wild type protein based on both size-exclusion chromatography and the interaction assay with MiD51 (Fig 1C, 1D and 1E). These results indicate that the interaction between MiD51 and Drp1 significantly depends on Drp1 oligomerization.

### Structural analysis of the cytoplasmic domain of MiD51

**Fig 2:**
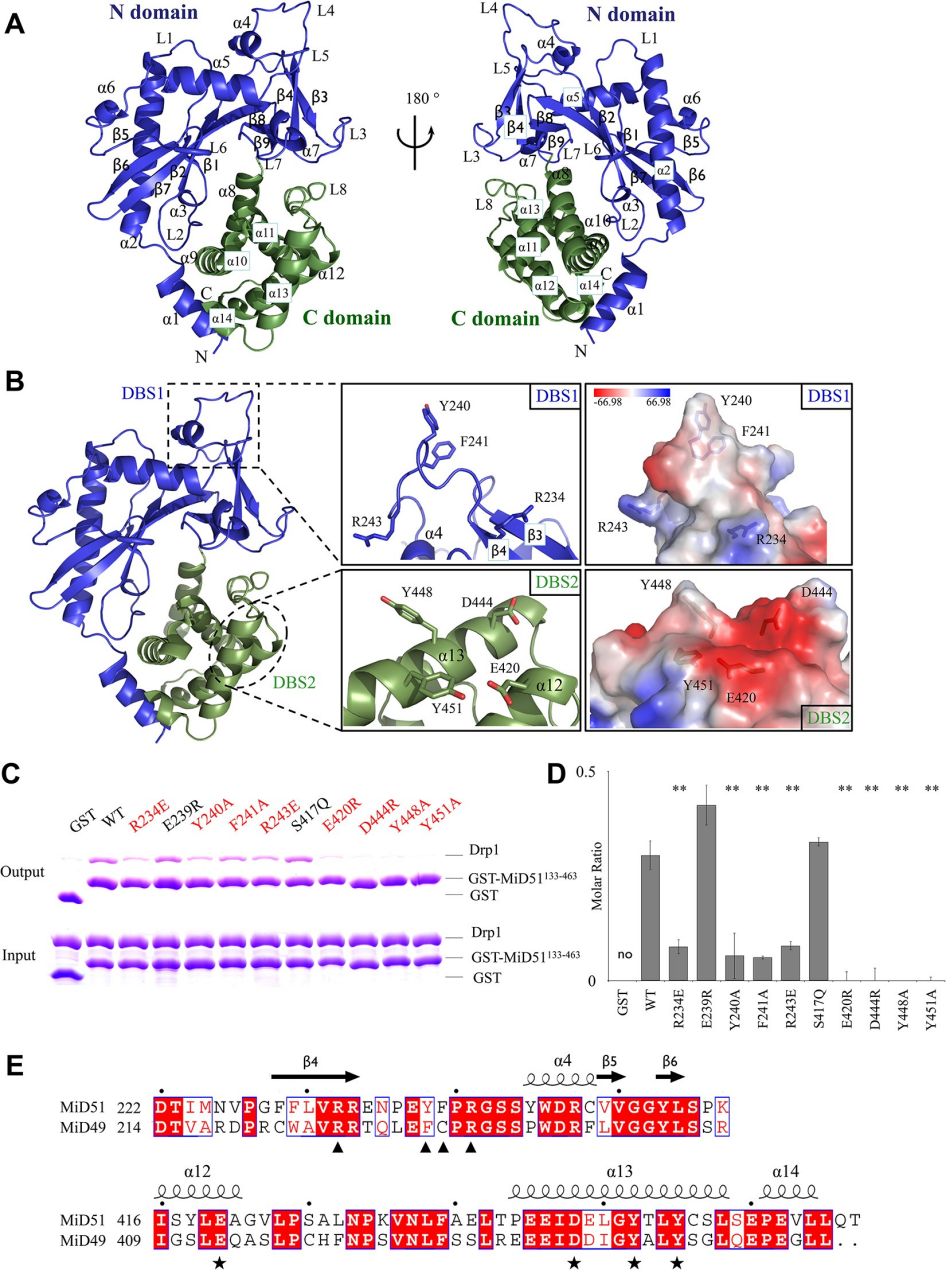
Two sites on MiD51 are involved in the interaction with Drp1. (A) Crystal structure of MiD51^133-463^. The N domain is colored blue, and the C domain is colored green. Secondary structure elements are labeled. (B) MiD51 sites that bind to Drp1. Left: Overview of the two MiD51 sites, which are outlined in dotted rectangles. Middle: Close-up views show the two binding sites. Key residues are labeled. Right: Electrostatic surface representation of two binding sites, with blue coloring indicating positive charges and red coloring indicating negative charges. (C) WT and mutant GST-MiD51^133-463^ in vitro pull-down assays were performed with purified Drp1. (D) Quantitation of the results in (C). The binding affinity is expressed as molar ratio of Drp1 to MiD51 mutants. Data are shown as mean ± SEM of three independent experiments performed in triplicate, with ** P < 0.005 compared to wild-type. (E) Sequence alignment of MiD51 and MiD49 sequences. Strictly conserved residues are highlighted in red. Secondary structural elements are depicted on the top of the alignments. Residues involved in Drp1 interaction are marked with★ for DBS1 and ▲ for DBS2.

To understand how MiD51 interacts with Drp1 during mitochondrial fission, we performed crystal structure studies and solved two types of MiD51 crystal structures under apo conditions (S1 Table). Type I contains the cytosolic domain MiD51^129-463^, and the crystal space group is *P*4_1_2_1_2 with one molecule per asymmetric unit. Type II contains the fragment MiD51^133-463^, which was expressed as a C-terminal 6×His fusion protein, and the crystal space group is *P*1 with two molecules per asymmetric unit. The overall structure consists of a central β-strand region flanked by two α-helical regions (Fig 2A) and looks similar to NTPase family crystal structures published by two groups [17,18]. The Type I and Type II crystal structures are almost identical, with a RMSD (root mean square deviation) variation of 1.14 Å for 329 aligned C_α_ atoms. By comparison, we found that all of the released crystal structures of MiD51[17,18] from PDB (S2 Table), which include different nucleotide forms (Apo, ADP or GDP), lack distinct conformational changes when compared to the Type I and Type II crystal structures, with RMSD variations ranging from 0.97 to 1.88 Å (S1B Fig and S3 Table). Such a small conformational change is probably due to different constructs, crystallization conditions and crystal packing. And the ADP/GDP binding sites are almost identical, which implies the structural rigidity of MiD51. It was reported that MiD51 can form dimers primarily based on the crystal packing of MiD51, but we did not observe such packing in our two crystal types (S1C Fig). Further studies are needed to determine the oligomer state of MiD51, and we describe these studies in a later section.

### Two sites on MiD51 are involved in the interaction with Drp1

The crystal structure of MiD51 supplies limited information about Drp1 binding. Therefore, we performed a systematic analysis of MiD51 mutants (S4 Table) to identify which region is involved in the interaction with Drp1. Initially we designed a series of mutant proteins, each containing a cluster of three or four mutated residues. We then used a pull-down assay to test the affinity of each MiD51 mutant for Drp1. These assays indicated that six MiD51 mutations disrupt the interaction with Drp1 (S2A and S2B Fig). Next, we did a second round of point mutations of MiD51. We found eight mutant proteins with decreased affinity for Drp1 (Fig 2C and 2D, S2C, S2D and S3B Fig). There was almost no conformational change in the mutant proteins compared to wild type MiD51 based on circular dichroism (CD) spectroscopy (S2E Fig).

We analyzed the distribution of these sites and found that the eight mutations are located in two areas. The first area contains four residues, R234, Y240, F241 and R243, which are located on an exposed loop between β4-α4 (Fig 2B). When these residues are substituted with alanine or glutamate (R234E, Y240A, F241A and R243E), the resulting mutant proteins have modest or serious decreases in Drp1 binding affinity. The decrease in Drp1 binding was confirmed using a pull-down assay and results were quantified (Fig 2C and 2D and S3B Fig). This suggests that the exposed loop is a main determinant for Drp1 binding. We name this area DBS1 (Drp1 Binding Site One) (Fig 2B), which is consistent with previous studies [17,18]. The second area contains the amino acids E420, D444, Y448 and Y451(Fig 2B). Mutation of these residues by substituting with alanine, or by substituting aspartate and glutamate with arginine (E420R, D444R, Y448A and Y451A), results in a more dramatic effect on the ability of MiD51 to bind Drp1, and in some cases even abolishes binding (Fig 2C and 2D, S3B Fig). We define this area as DBS2 (Drp1 Binding Site Two), which is located on α12 and α13 in the C domain and forms a surface for Drp1 binding (Fig 2B). Therefore, MiD51 requires DBS2, a surface in the C domain, to cooperate with Drp1 binding. An amino acid sequence alignment of MiD51 and MiD49 proteins from different species reveals that these eight DBS1 and DBS2 residues are highly conserved, except that F241 is strictly conserved in MiD51 but not in MiD49 (Fig 2E and S2F Fig). Based on the crystal structure of MiD49[18], these eight residues also form an exposed loop in the N domain and a surface in the C domain for Drp1 binding.

### MiD51 forms a dimer and is important for mitochondrial dynamics

**Fig 3:**
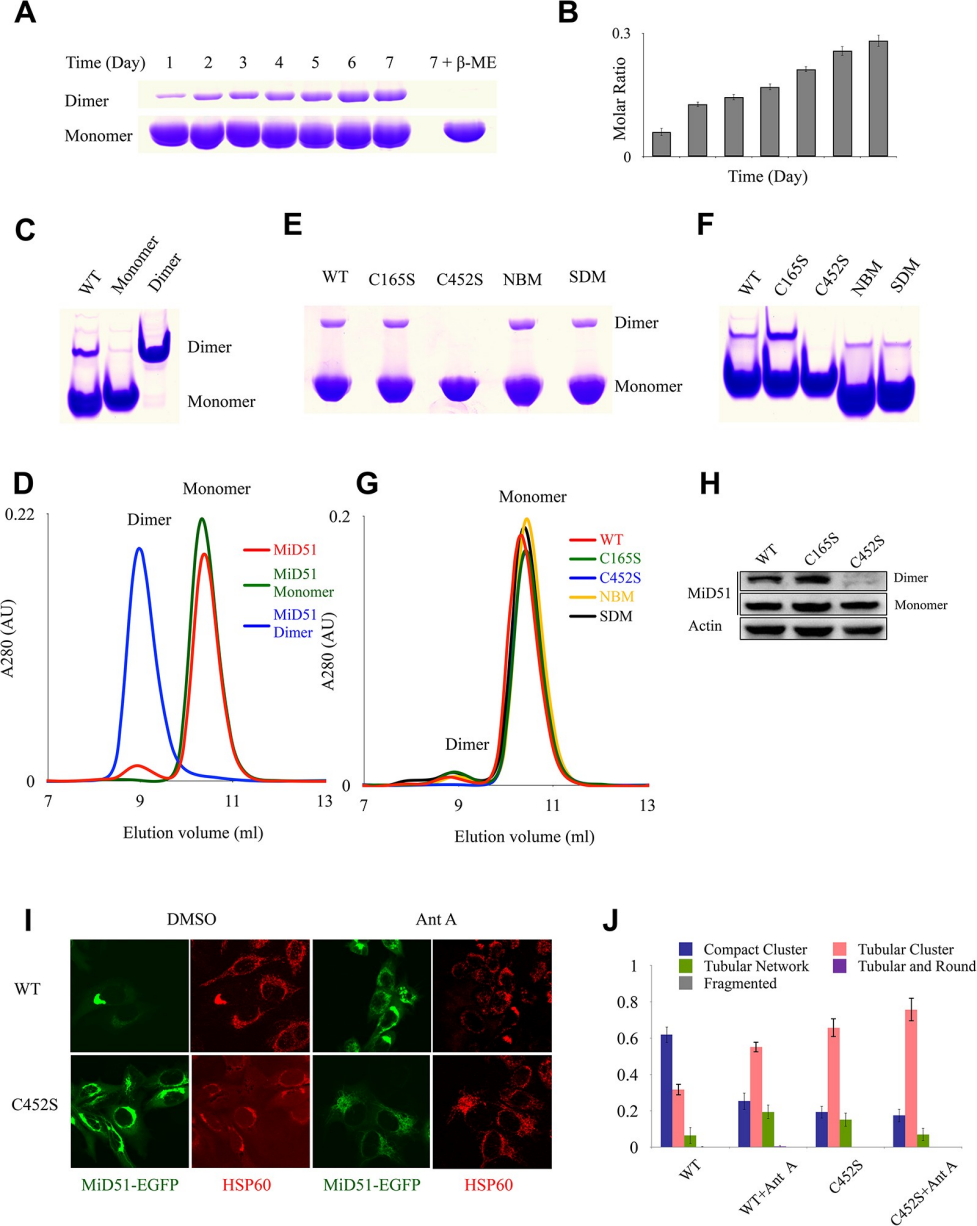
MiD51^133-463^ forms a dimer via an interface close to residue C452 and is important for its interaction with Drp1. (A) A time course experiment, where the level of dimer formation was quantified every twenty four hours, and non-reducing SDS-PAGE indicates that MiD51^133-463^ can form dimers in the air. (B) Quantification of the results in (A). The level of dimerization is expressed as the ratio of dimer to monomer. All error bars represent SD from three independent experiments. (C) Native PAGE analysis of monomeric and dimeric MiD51^133-463^. (D) Size-exclusion chromatography analysis of monomeric and dimeric MiD51^133-463^. Size-exclusion chromatography was performed with the size-exclusion column Superdex 75 PC 3.2/20 (GE Healthcare). The blue profile represents dimer and the green profile represents monomer. (E), Non-reducing SDS-PAGE of wild-type and mutant MiD51^133-463^ shows that the C452S mutant is not able to form dimers. (F) Native PAGE of wild-type and mutant MiD51^133-463^ also indicates that the C452S MiD51^133-463^ mutant does not form dimers. (G) Gel filtration analysis of MiD51^133-463^ and mutants shows that the dimer peak is missing in the C452S mutant. (H) Dimerization analysis of full-length MiD51 and mutants C165S and C452S in HeLa cells. Wild type or mutant MiD51-Myc was expressed in HeLa Cells and analyzed with Myc antibody. Actin is a loading control. (I) HeLa cells were transfected with wild type MiD51 or mutant C452S, and treated with Antimycin A. Transfection with wild type MiD51 results in mitochondrial fission, but no fission is observed in cells transfected with the C452S mutant, indicating that in the absence of dimer formation, MiD51 can not perform its function in mitochondrial fission. (J) Quantification of the results in (I). Mitochondrial morphology was scored as described previously [26]. Data were obtained from three independent experiments, with 100 cells per experiment, ** P < 0.005.

Mitochondrial fission receptors, such as Fis1 and Mff, form dimers to perform their functions in mitochondrial fission [12]. A previous study reported that MiD51 could form a dimer under non-reducing conditions [26], suggesting that the region of residues 49 to 195 is responsible for MiD51 dimerization [26]. But based on the crystal structure, another study found that MiD51 forms a dimer via electrostatic interactions in the N-terminal helix, and the dimerization is very important for its function in mitochondrial fission[18]. Surprisingly, we did not observe a similar surface mediating the dimerization of MiD51 in our crystal packing. Therefore we experimentally determined whether MiD51 forms dimers. Using a time course assay where the level of dimer formation was quantified every twenty-four hours, we determined that MiD51^133-463^ does form dimers and that the level of dimerization continues to increase over time (Fig 3A and 3B). These results correlate well with the results of Zhao et al[26]. However, a limited amount of MiD51^133-463^ protein exists as dimers based on native-PAGE and size-exclusion chromatography (Fig 3C and 3D), indicating that the majority of MiD51^133-463^ exists as a monomer.

To determine which residue mediates MiD51 dimerization, we analyzed the MiD51 sequence and found that there are seven cysteines, but only two, C165 and C452, are exposed on the protein surface. When C452 was substituted by serine (C452S) the MiD51 protein lost its ability to form dimers, as shown by lack of dimer formation in non-reducing PAGE, Native PAGE and size-exclusion chromatography (Fig 3E,3F and 3G). In contrast, another cysteine mutant C165S showed dimerization similar to wild type (Fig 3E, 3F and 3G). This suggests that C452, not C165, is the residue that is on or close to the dimerization interface of MiD51. We also found that the supposed dimer surface mutant [18] (SDM, R169A/R182A/D183A/Q212A/N213A) and nucleotide binding site mutant (NBM, H201D/R342E/K368E) (S1A Fig) were still capable of dimerization similar to wild type (Fig 3E, 3F and 3G). Therefore, the dimerization of MiD51 is not mediated by the previously supposed dimer surface[18] or the nucleotide binding site. Thus, the results certify that the interface close to C452 is required for dimerization of MiD51.

Next we tested whether MiD51 dimerization *in vivo* plays a critical role in mediating mitochondrial dynamics. When MiD51 wild type, C165S and C452 mutants expressed in HeLa cells, dimerization of the C165S mutant protein was the same as wild type, but no dimerization of the C452S protein was observed (Fig 3H). When overexpressed in HeLa cells, EGFP-MiD51^WT^ caused mitochondrial clustering as reported [15]. However, in HeLa cells overexpressing EGFP-MiD51^C452S^, the level of mitochondrial clustering was greatly alleviated compared to wild type (Fig 3I and 3J), suggesting the role of MiD51 dimerization in mitochondrial dynamics. In MiD51-overexpressing cells, treatment with Antimycin A (an inhibitor of complex III of the electron transport chain) induces rapid mitochondrial fission [18]. So we used Antimycin A to assess the mitochondrial fission activity of MiD51 wild type and C452S mutant. When expressed in HeLa cells in the presence of Antimycin A, EGFP-MiD51^WT^ alleviated mitochondrial aggregation and changed the mitochondria to a tubular morphology; however, the C452S mutant protein does not induce changes in mitochondrial morphology (Fig 3I and 3J), indicating that it is also defective in fission activity. Therefore, we conclude that C452 is closely associated with the dimerization of MiD51 *in vivo*, and the dimerization is required for normal mitochondrial dynamics.

## Discussion

The role of MiD51 in mitochondrial fission has been well established [15,17,26–29]. MiD51 mediates mitochondrial fission by recruiting Drp1 to the outer mitochondrial membrane and regulating its assembly and mitochondrial fission activity in a GTP-dependent manner. We have elucidated in molecular detail how the interaction between MiD51 and Drp1 is regulated by multi-faceted mechanism.

The changes in Drp1 conformation and oligomerization upon GTP binding, hydrolysis and release, is associated with the procession of mitochondrial fission [30]. We suggest that the interaction between MiD51 and Drp1 undergoes changes during this process. Initial insight came from the binding of MiD51 for Drp1 under different nucleotide binding states. We determined that MiD51 binds effectively to the GTP and GMP-PNP bound states of Drp1, which depends on the polymerization of Drp1. Recent research suggests that oligomerization of Drp1 is required for its interaction with Mff, whereas MiD51 does not have a strong requirement for Drp1 oligomerization because AAAA mutant form of Drp1, which are only capable of forming dimers, still show binding activity for MiD51 [31]. Although the AAAA mutant has the capacity to bind MiD51, its binding affinity is reduced compared to wild type as we showed (Fig 1A–1D).

We then gained significant insight into the interaction between MiD51 and Drp1 by examining the contact interface on MiD51. By performing a systematic screen of proteins with mutations in surface residues, we determined that two regions, DBS1 and DBS2, in MiD51 make direct contact with Drp1. DBS1, containing R234, Y240, F241 and R243, is located on an exposed loop of the N domain. The location of this binding site is consistent with previous studies [17], one of which proposed that the topology of the loop is a critical factor for Drp1 binding. This suggests that electrostatic interactions and hydrophobic interactions may play important roles in the binding of Drp1 to MiD51. DBS2 is a novel region located on the surface of the C domain. Single mutations, such as E420R, D444R, Y448A and Y451A, completely abolish MiD51-mediated binding of Drp1, suggesting that DBS2 is much more important than DBS1 for Drp1 recruitment. We know that the interaction between MiD51 and Drp1 changes during the process of mitochondrial fission, so the interaction may need more than one binding site between MiD51 and Drp1. In addition to the exposed loop in N domain, we have determined that another region in MiD51 makes direct contact with Drp1, and the residues are highly conserved between MiD51 and MiD49. It seems likely that the two binding regions on MiD51 are responsible for the complicated interaction with Drp1 during the process of mitochondrial fission. But the precise role of MiD51 in Drp1 polymerization and mitochondrial fission still remains elusive.

We also determined that MiD51 forms dimers through an interface close to residue C452 located in the C terminal region, although the majority of MiD51 protein exists as monomer. It is easily to associate with disulfide bond formation when residue cysteine is important for dimerization. But it seems that MiD51 is not the same case, considering cytosol is a reducing environment, so we could only conclude that C452 is closed to the dimer interface of MiD51 and leave an open question whether the disulfide bond forms via the residue C452 in vivo. The monomer-dimer state of MiD51 is closely related to its interaction with Drp1 and regulates mitochondrial fission activity, which could be reflected by different metabolism state of cells. We note that the Drp1 receptor Mff exits as a tetramer formed via its coiled coil region, and only multimeric Mff can bind Drp1 effectively, facilitate assembly of Drp1 polymer, stimulate GTPase activity and trigger mitochondrial fission [18,32]. So the MiD51 and Mff receptors function in a similar way by forming a dimer or tetramer to recruit Drp1 and regulate mitochondrial fission. However, the residue C452 required for dimerization of MiD51 is not conserved in MiD49, so MiD51 and MiD49 might mediate mitochondrial fission via different regulatory mechanisms. Further work will be necessary to understand whether and how MiD49 forms dimers to regulate fission.

**Fig 4:**
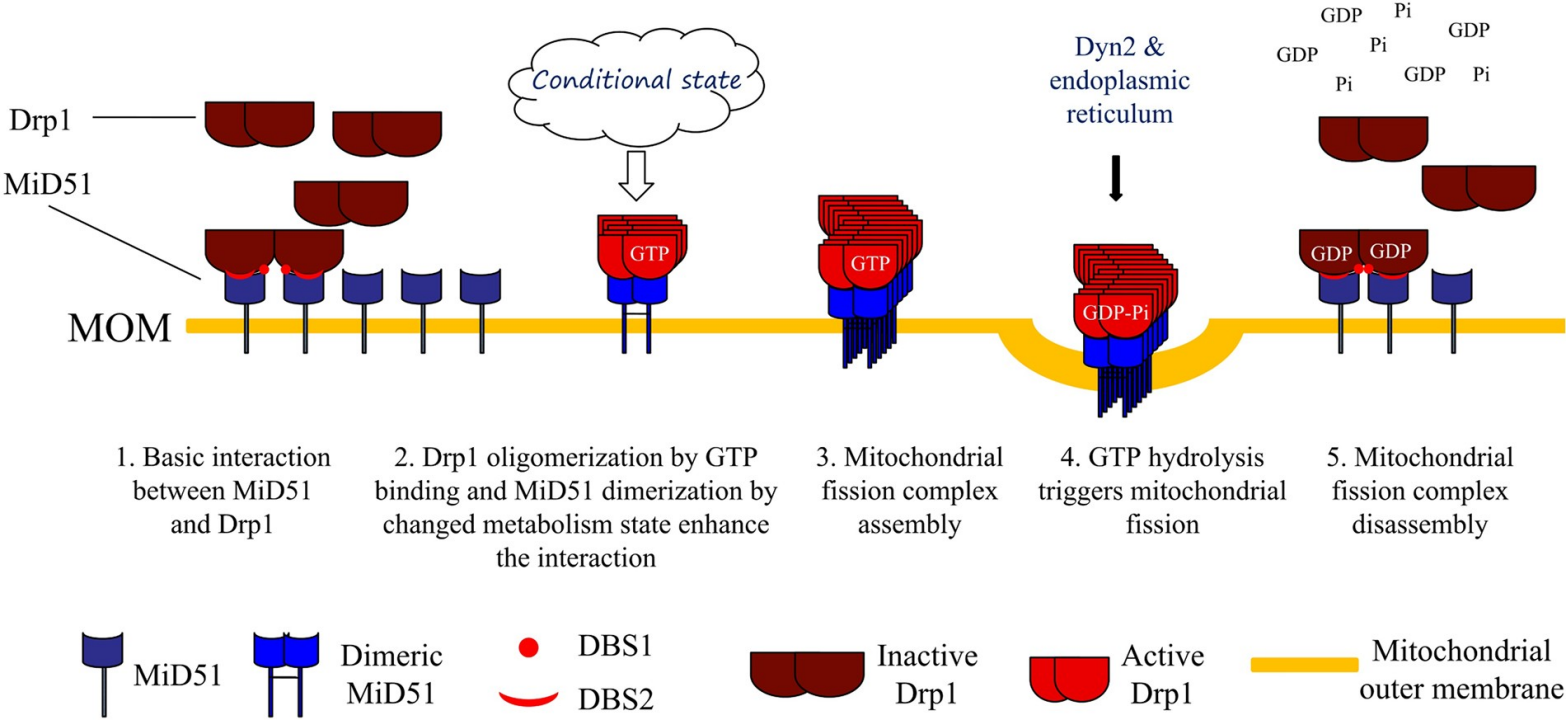
A proposed model for MiD51-mediated recruitment of Drp1 and mitochondrial fission.

Collectively, we propose a model for MiD51-mediated recruitment of Drp1 to regulate mitochondrial fission (Fig 4). **1**. At basic conditions, most Drp1 protein is inactive in the cytoplasm, and MiD51 doesn’t form dimers; therefore, only a small amount of Drp1 binds to MiD51; 2. For mitochondrial fission, Drp1 binds to GTP and undergoes oligomerization and MiD51 forms dimers, leading to the enhancement of the interaction between MiD51 and Drp1 by DBS1 and DBS2; 3. Dimeric MiD51 recruits more oligomeric Drp1 to the mitochondrial outer membrane, resulting in the formation of the mitochondrial fission complex around the fission site; 4. GTP hydrolysis further enhances the interaction between MiD51 and Drp1, and triggers mitochondrial fission by the fission complex and other factors such as Dyn2 and endoplasmic reticulum; 5. After mitochondrial fission is complete along with the production of GDP, oligomeric Drp1 depolymerizes, the interaction between MiD51 and Drp1 is weakened to that observed at basic levels, and finally Drp1 is released from the membrane and localizes to cytoplasm where it is free to function in another cycle of mitochondrial fission.

## Materials and methods

### Molecular cloning and plasmid constructions

The open reading frames (ORF) encoding MiD51^ΔTM^, MiD51^129-463^, MiD51^133-463^ and mutants were amplified by PCR from the full-length human MiD51 ORF (GenBank accession No. NM_019008) and cloned into the pGEX-6P-1 vector (GE Healthcare), or pET22b (Novagen) derivative vector with a N-terminal 6*His tag. Drp1 (GenBank accession No. NM_005690) and its mutants were cloned into the pET22b (Novagen) derivative vector with a C-terminal 6*His tag. All site-directed mutagenesis of MiD51 and Drp1 were performed by the overlapping PCR method.

### Protein expression and purification

All constructs of MiD51 were expressed in *Escherichia coli* BL21 (DE3) (Invitrogen). Recombinant proteins were induced by addition of 0.3 mM IPTG at a culture density of OD600~0.6, followed by 16 h incubation at 16°C. To purify His tag proteins, the bacterial cells were lysed by high-pressure homogenization in lysis buffer containing 50 mM Tris-HCl, 300 mM NaCl, 40 mM imidazole, pH 8.0. Cell debris was removed by centrifugation at 16,000 g for 40 min, the supernatant was applied to Ni-NTA Sepharose (GE Healthcare) and washed with lysis buffer, and the protein was eluted with lysis buffer plus 300 mM imidazole and concentrated using Amicon Ultra-4 centrifugal filter units (10 kDa cutoff, Millipore). A HiTrap Desalting column (5 ml, GE Healthcare) was used to change the buffer of proteins to 20 mM Tris-HCl pH 8.0, 50 mM NaCl. The protein was further purified by anion-exchange chromatography on a Resource Q column (GE Healthcare) with a NaCl linear gradient of 50–600 mM in 20 mM Tris-HCl pH 8.0. The eluted fractions containing MiD51 were pooled and concentrated, and finally purified by size-exclusion chromatography using a Superdex 75 column (GE Healthcare) pre-equilibrated with 20 mM HEPES pH 7.5, 150 mM NaCl.

To purify GST fusion proteins, bacterial cells were lysed in lysis buffer (10 mM Na2HPO4, 1.8 mM KH2PO4, 140 mM NaCl, 2.7 mM KCl, 1 mM DTT pH 7.4), and the supernatant was applied to Glutathione Sepharose (GE Healthcare) loaded into a 20-ml gravity flow column. For crystallization, the resins were first washed with the lysis buffer, then the GST fusion proteins were digested using PreScission protease (GE Healthcare) at 16°C overnight on column. The digested MiD51 protein was eluted using the lysis buffer. The next steps were the same as His tag proteins. For GST pull-down assaay, GST-fused proteins were directly eluted with 20 mM reduced glutathione, 50 mM Tris-HCl pH 8.0, 150 mM NaCl. After concentration, GST-fused protein was directly changed the buffer to 20 mM HEPES pH 7.5, 150 mM NaCl. The selenomethionine (SeMet)-labeled MiD51^129-463^ was prepared as described previously [33,34]. In brief, the expression vector containin GST-fused MiD51^129-463^ was transformed into the methionine auxotroph *E*. *coli* B834 strain (Novagen). The cells were grown in M9 medium supplemented with YNB medium, 50 g/L glucose, 2 mM MgSO_4_, 0.1 mM CaCl_2_ and 50 mg/L of L-selenomethionine. The purification process of the SeMet-labeled MiD51^129-463^ was the same as that used for the native protein.

Wild type Drp1 and its mutants were expressed in *E*. *coli* Rosseta (DE3) cells (Invitrogen), and the expression and purification process was in the same way as MiD51^133-463^ with 6*His tag.

### GST pull-down assay

For GST pull-down assay, equal amounts of GST, GST-fused MiD51^133-463^, and GST-fused mutant proteins were loaded onto 15 μl of Glutathione Sepharose 4B slurry beads in assay buffer (20 mM HEPES, pH 7.5, 150 mM NaCl, 1% Triton X-100, 1mM MgCl_2_). After incubation with equal molar of Drp1 for 3 h at 4°C, the pellets were washed three times with 500 μl of assay buffer, subsequently incubated with SDS-PAGE sample buffer at 95°C, separated on 12% SDS-PAGE gels and detected using Coomassie blue staining. In the case of pull-down assay with different nucleotides (GTP, GMP-PNP, GDP-AlFx or GDP), MiD51 and Drp1 were mixed evenly first before dispensing the same amount of volume to the same amount of resin, and then 1 mM nucleotide at final concentration was added in the assay buffer and wash buffer.

### Crystallography

Crystals of MiD51^133-463^ and Se-MiD51^129-463^ were obtained using the hanging drop vapor diffusion method at 16°C. To set up a hanging drop, 1 μl of concentrated protein solution was mixed with 1 μl of crystallization solution. The final optimized crystallization condition was 0.6 M NaH_2_PO_4_/K_2_HPO_4_, pH 7.0 for MiD51^133-463^ at 30mg/ml and 0.2 M L-Proline, 0.1 M HEPES pH 7.0, 6% PEG3350 for MiD51^129-463^ at 18mg/ml. Before X-ray diffraction, crystals were soaked in crystallization solution containing 20% glycerol for cryo-protection. The diffraction data for native and SeMet derivative crystals were collected at 100 K at beamline BL17U at Shanghai Synchrotron Radiation Facility (SSRF). The diffraction data were processed and scaled using HKL2000 [35]. The structure of MiD51^129-463^ was solved with the single-wavelength anomalous diffraction (SAD) method. Selenium atoms were successfully located with the SHELXD [36] in HKL2MAP [37]. Phases were calculated and refined with SOLVE and RESOLVE [38,39]. An initial model was built using COOT [40] and further refined using REFMAC5 [41]. The structure of MiD51^133-463^ was solved by molecular replacement with Phaser [42] and further refined using REFMAC5. The stereo-chemical quality of the final model was validated by PROCHECK [43] and MolProbity [44]. The statistics for data processing and structure refinements are listed in S1 Table. All structural figures were prepared with PyMOL (http://www.pymol.org/).

### Circular dichroism (CD) spectroscopy

For CD spectroscopy assay, the wild type and mutant 6×His-MiD51^133-463^ protein samples were diluted to 0.2 mg/ml in buffer containing 10 mM Na_2_HPO4, 1.8 mM KH_2_PO4, 140 mM NaCl, 2.7 mM KCl, 1 mM DTT, pH 7.4. The spectra were recorded over the wavelength from 200 nm to 260 nm with a bandwidth of 1 nm and 0.5 s per step by using CD spectrometer (Chirascan-plus, Applied photphysics). All the measurements were repeated three times and the spectrum data were corrected by subtracting the buffer control.

### Immunofluorescence assay

For imaging, HeLa cells were grown to 60% confluence on coverslips and transfected with an equal amount of pEGFP-N1-MiD51 plasmids using PEI (Polyethyleneimine). About 24 h post-transfection, cells were washed twice with PBS, fixed with freshly prepared 3.7% (vol/vol) paraformaldehyde in PBS at 37°C for 15 min, and permeabilized with 0.2% Triton X-100 in PBS at 4°C for 10 min. Cells were incubated with primary antibodies as follows: rabbit monoclonal anti-HSP60 and mouse monoclonal anti DLP1 for 2 h at room temperature. After washing twice with PBS, primary antibodies were labeled with goat anti-mouse Alexa Fluor 647- and donkey anti-rabbit Alexa Fluor 555-conjugated secondary antibodies for 45 min. Cell images were acquired using 100× oil objective on a FV1000 OLYMPUS confocal microscope. All quantifications were done three times, and 100 cells were scored per experiment. Antimycin A was added at 10 mM in cell culture and treatment occurred for 1 hour before cells were fixed.

### Western blot assay

HeLa cells were transiently transfected with pcDNA3-c-5×Myc-MiD51 plasmids using PEI (Polyethyleneimine). About 24 h post-transfection, the cells were harvested and lysed in 1 ml of lysis buffer (50 mM Tris—Cl pH 7.5, 150 mM NaCl, 1 mM EDTA, 1% NP-40) containing 1 mM PMSF and protease inhibitor cocktail (Roche Applied Science) for 30 min on ice. After 17,000 g centrifugation for 10 min, the supernatant was added directly with laemmli buffer without reducing agent and heated for 5 min at 95°C and analyzed by 12% SDS-PAGE followed by Western blot.

## Supporting information

S1 Fig Comparisons between Mid51 protein crystal structures. (A) Topology of MiD51 and key residues involved in nucleotide binding, dimerization and Drp1 binding. Domain boundaries are marked with residue numbers. The NTPase domain can be divided into two sub-domains, N domain (133–339) and C domain (340–463). TM, transmembrane domain; IMS, inter-membrane space; MOM, mitochondrial outer membrane. (B) Comparison of the crystal structure of MiD51133-463 with the cytoplasmic domain crystal structure of MiD51 from PDB (codes 4OAF, 4OAG, 4OAH, 4NXT, 4NXV, 4NXU, 4NXW and 4NXX), and comparison of the crystal structure of MiD51129-463 with the crystal structure of MiD51 from PDB (code 4OAI). (C) Crystal packing of the MiD51 structures shown in (B).
(PDF)

S2 Fig Systematic mutation screening to investigate the regions of Mid51 that are involved in the interaction with Drp1. (A) Mutant forms of MiD51 containing clusters of three or four mutated residues were initially tested for ability to bind Drp1 with in vitro GST pull-down assays. Six MiD51 mutants that disrupt the interaction with Drp1 are colored in red. (B) Quantification of the results in (A). The binding affinity is expressed as molar ratio of Drp1 to MiD51 mutants. Data are shown as mean ± SEM of three independent experiments performed in triplicate, with ** P < 0.005 compared to wild-type. (C) In vitro GST pull-down assays were used to screen the single point mutants based on the results of (A) and (B). Mutations that disrupt the interaction with Drp1 are colored in red. (D) Quantification of the results in (C). The binding affinity is expressed as molar ratio of Drp1 to MiD51 mutants. Data are shown as mean ± SEM of three independent experiments performed in triplicate, with ** P < 0.005 compared to wild-type. (E) Circular dichroism spectroscopy confirmed that MiD51 mutants that have disrupted interactions with Drp1 still have the same conformation as wild type. (F) Sequence alignment of full-length MiD51 and MiD49 proteins. MiD51 and MiD49 proteins are distinguished by grey shading. Strictly conserved residues are highlighted in red, and moderately conserved residues are outlined in blue. Residues involved in Drp1 interaction are marked with ★ for DBS1 and ▲ for DBS2. The secondary structures are shown above the sequences.
(PDF)

S3 Fig Original gel photos for SDS-PAGE. (A) Pull-down assays were performed to test the binding of purified Drp1 or mutants to GST-MiD51^133-463^ in the presence of different nucleotides, corresponding to Fig 1A. (B) WT and mutant GST-MiD51^133-463^ in vitro pull-down assays were performed with purified Drp1, corresponding to Fig 2C.
(PDF)

S1 Table Data collection and refinement statistics. (DOCX)

S2 Table Sum of partial crystallographic statistics for MiD51129-463, MiD51133-463, and released PDB crystal structures. (DOC)

S3 Table RMSD variations for superimposition of the Cαbackbone of MiD51129-463, MiD51133-463, and released PDB crystal structures. (DOC)

S4 Table Mutation screening of residues on MiD51 interacting with Drp1. (DOC)
