# Correction: New interfaces on MiD51 for Drp1 recruitment and regulation

**DOI:** 10.1371/journal.pone.0217720

**Published:** 2019-05-28

**Authors:** Jun Ma, Yujia Zhai, Ming Chen, Kai Zhang, Quan Chen, Xiaoyun Pang, Fei Sun

The following information is missing from the Data Availability statement: The coordinates for the crystal structures of MiD51^129-463^ and MiD51^133-463^ have been deposited in the Protein Data Bank (PDB), with the accession codes 5X9B and 5X9C, respectively.
