# Supplementary figures and images for: New interfaces on MiD51 for Drp1 recruitment and regulation
Source: PLoS One. 2019 Jan 31;14(1):e0211459. doi: 10.1371/journal.pone.0211459 (PMC6355003; doi:10.1371/journal.pone.0211459)

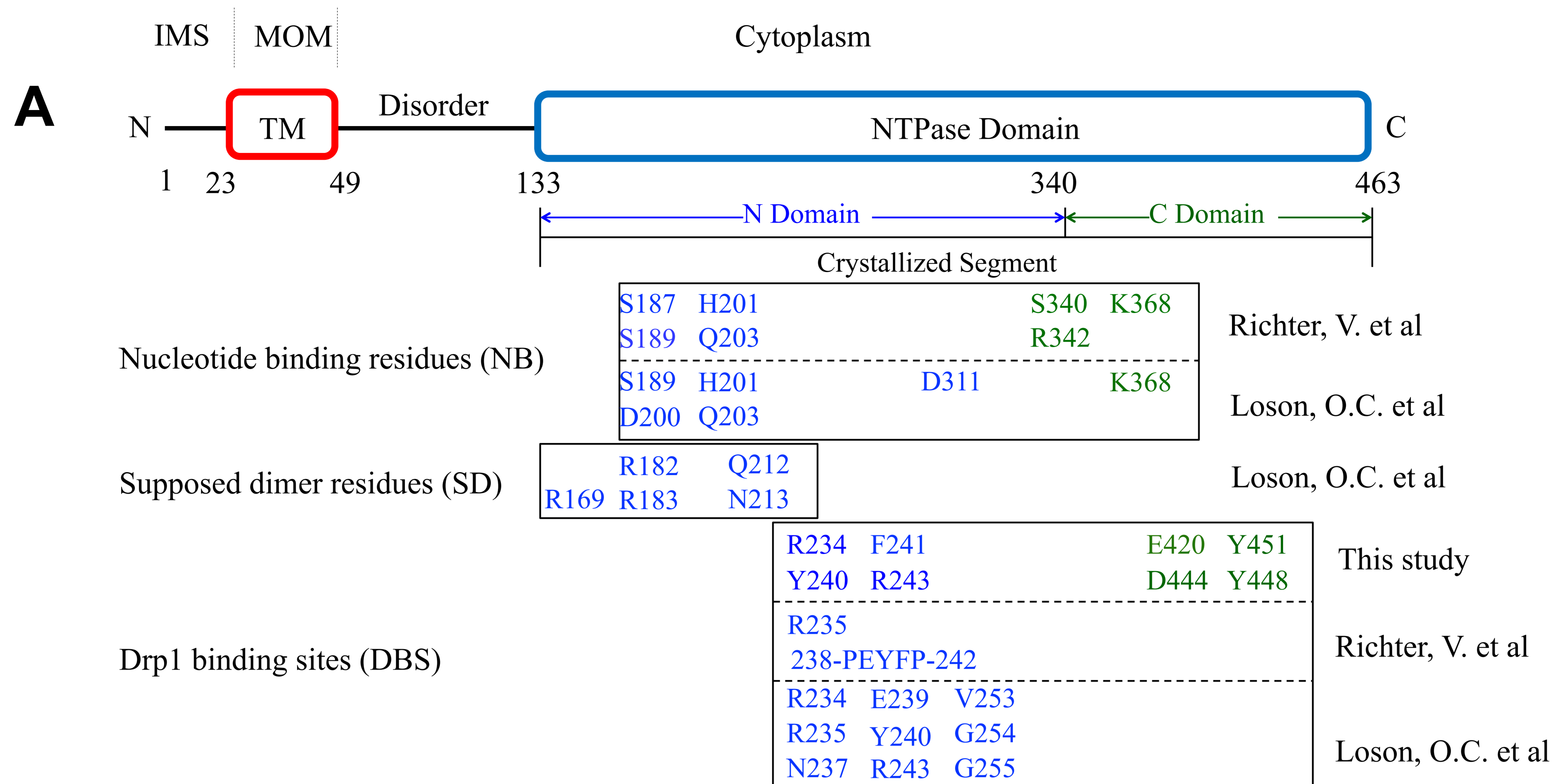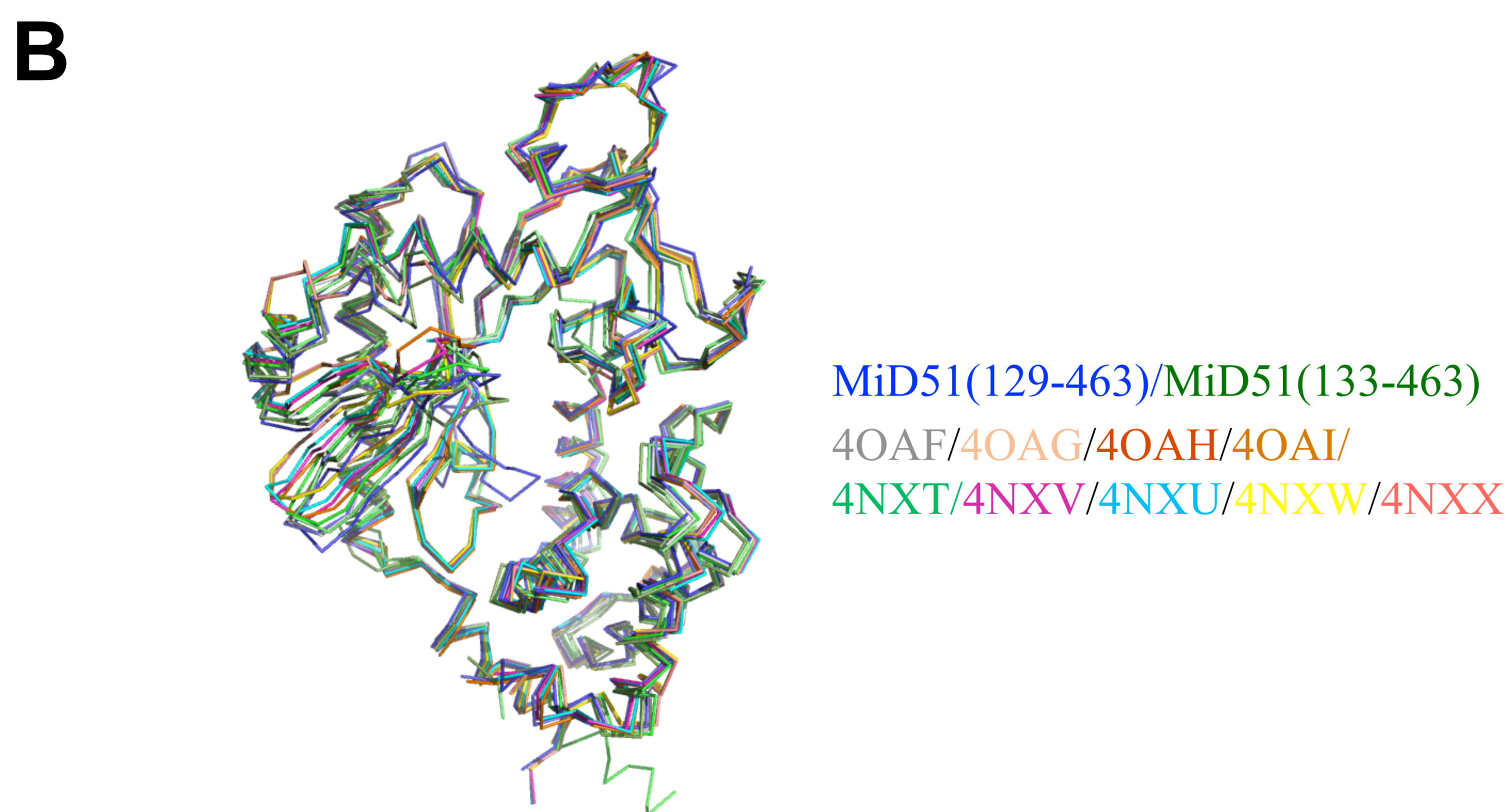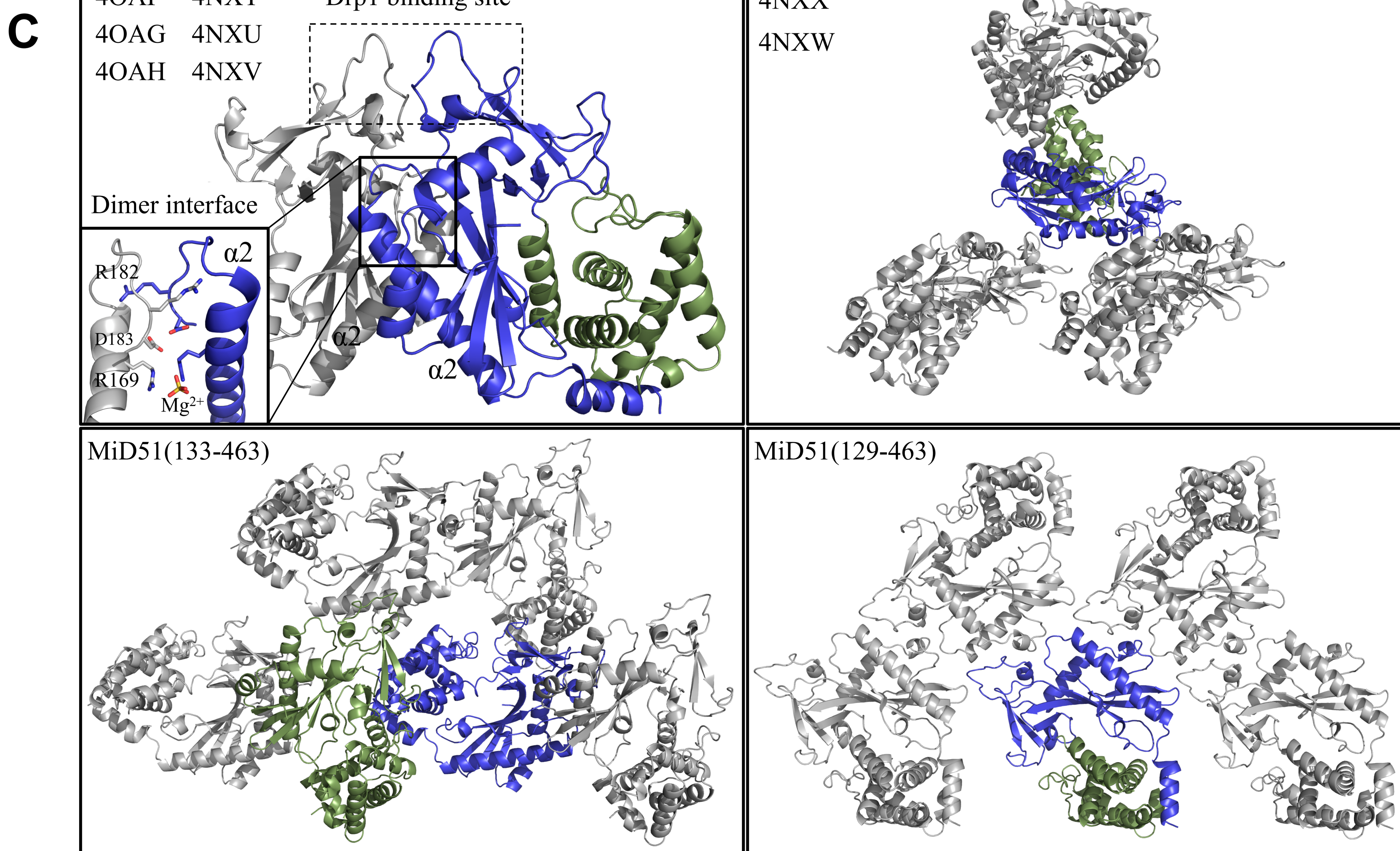

Supplement: S1 Fig — (A) Topology of MiD51 and key residues involved in nucleotide binding, dimerization and Drp1 binding. Domain boundaries are marked with residue numbers. The NTPase domain can be divided into two sub-domains, N domain (133–339) and C domain (340–463). TM, transmembrane domain; IMS, inter-membrane space; MOM, mitochondrial outer membrane. (B) Comparison of the crystal structure of MiD51133-463 with the cytoplasmic domain crystal structure of MiD51 from PDB (codes 4OAF, 4OAG, 4OAH, 4NXT, 4NXV, 4NXU, 4NXW and 4NXX), and comparison of the crystal structure of MiD51129-463 with the crystal structure of MiD51 from PDB (code 4OAI). (C) Crystal packing of the MiD51 structures shown in (B). (PDF) [file pone.0211459.s001.pdf]

**A**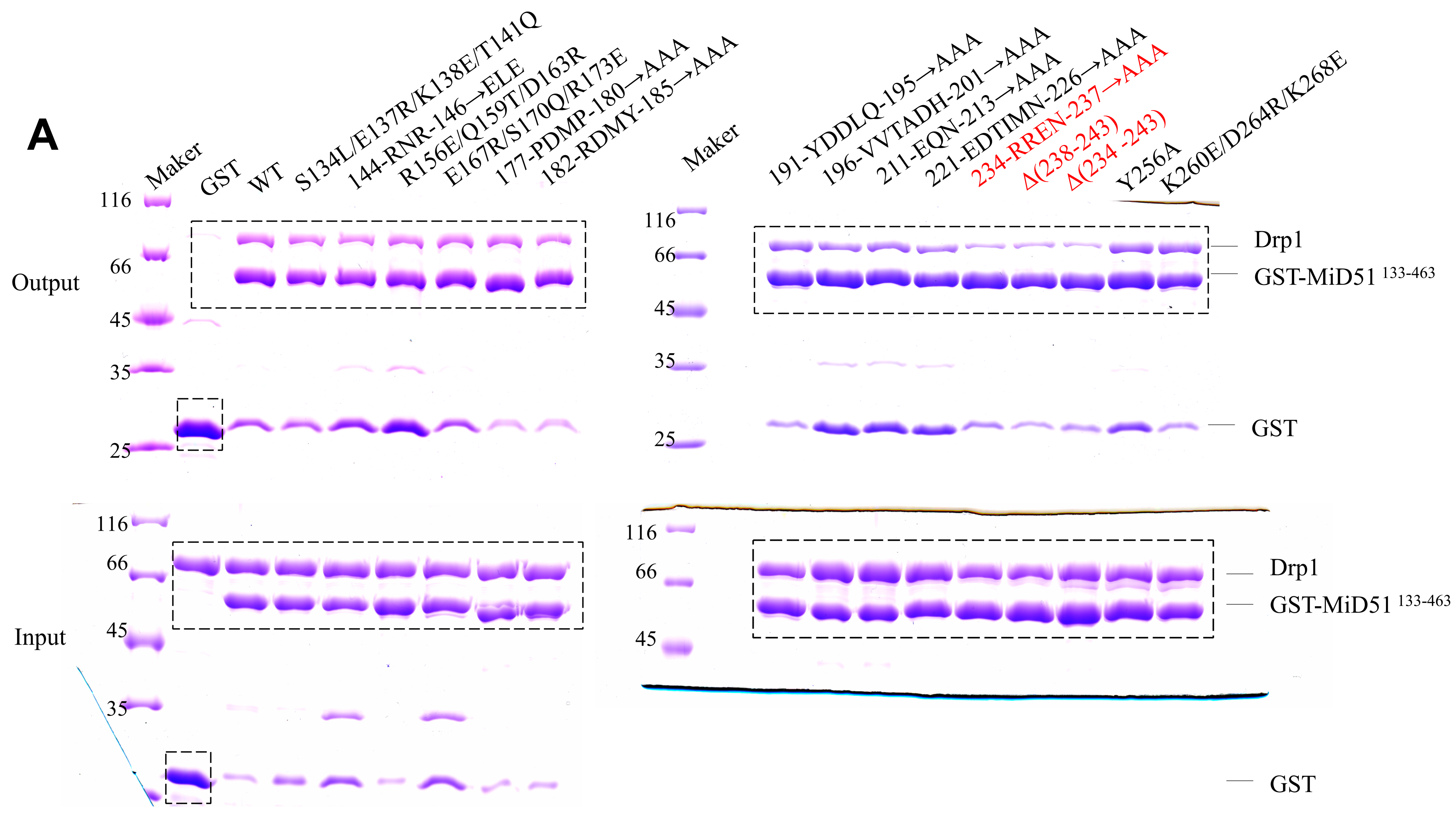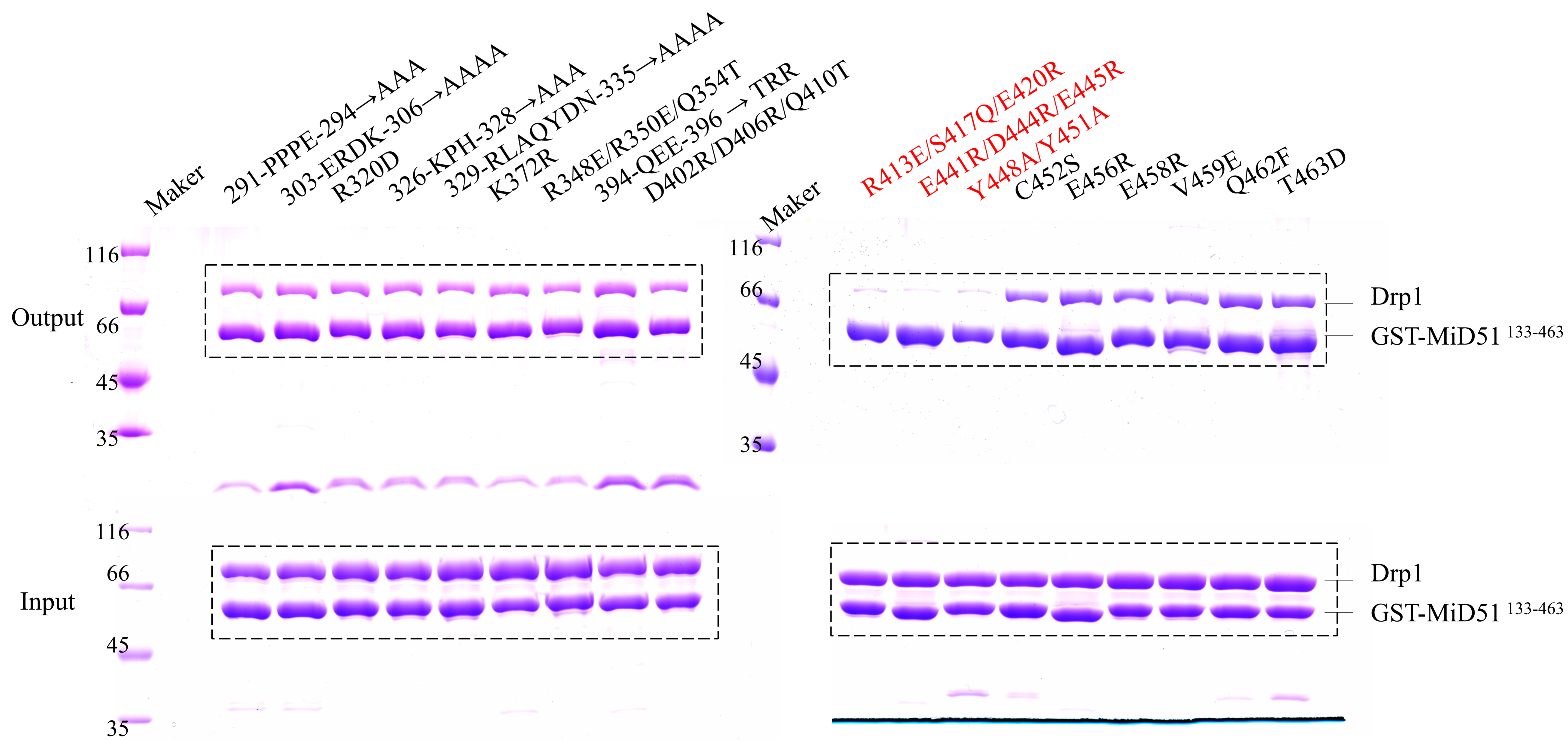

**B**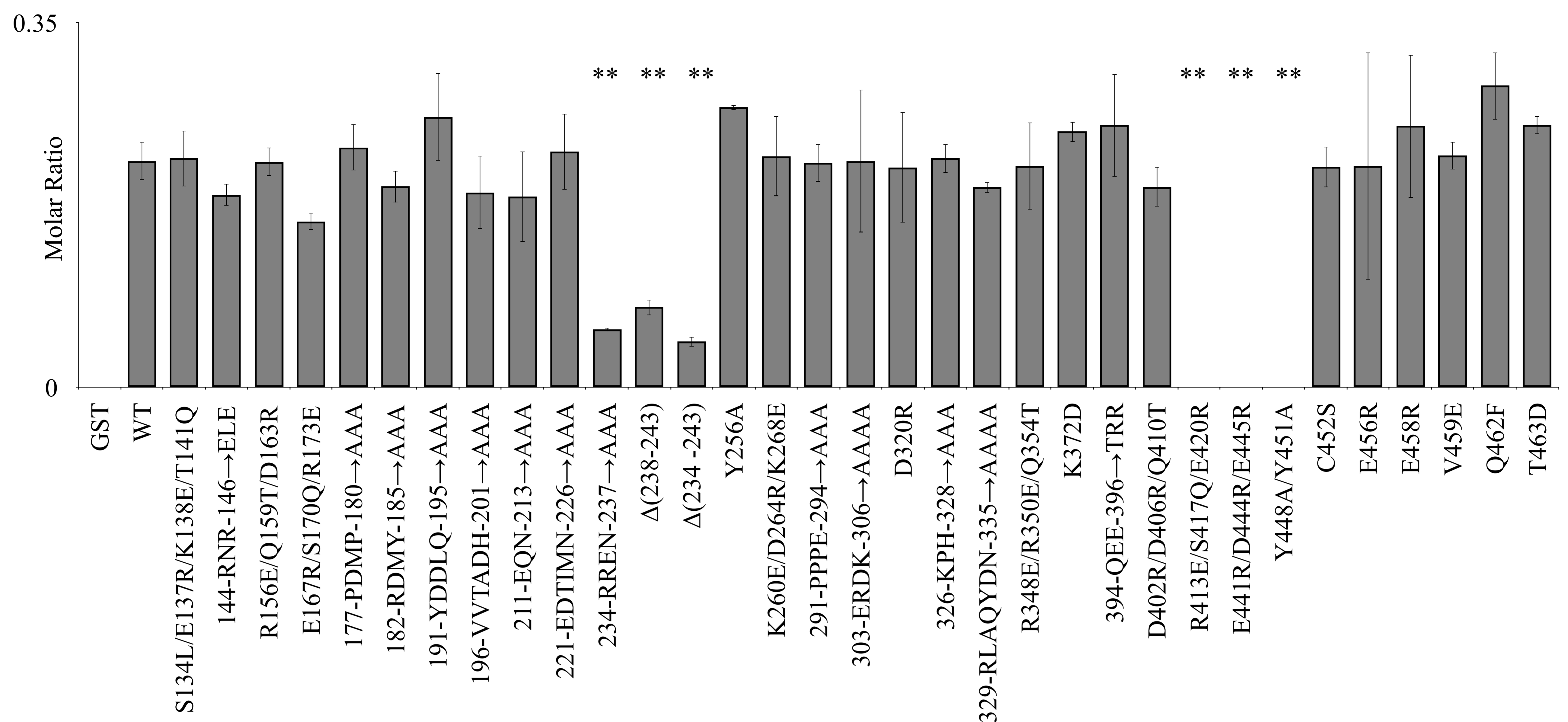**C**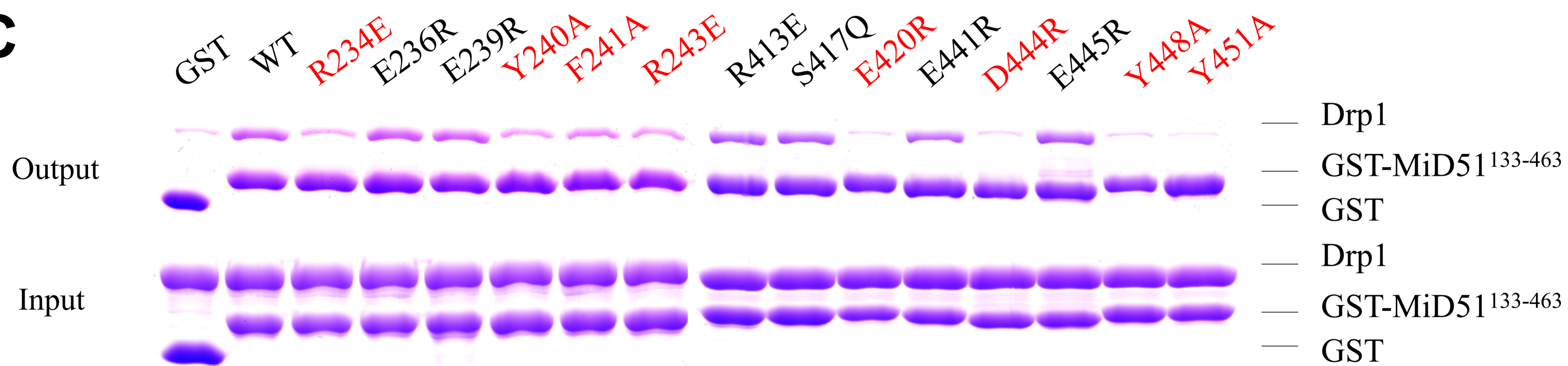**D**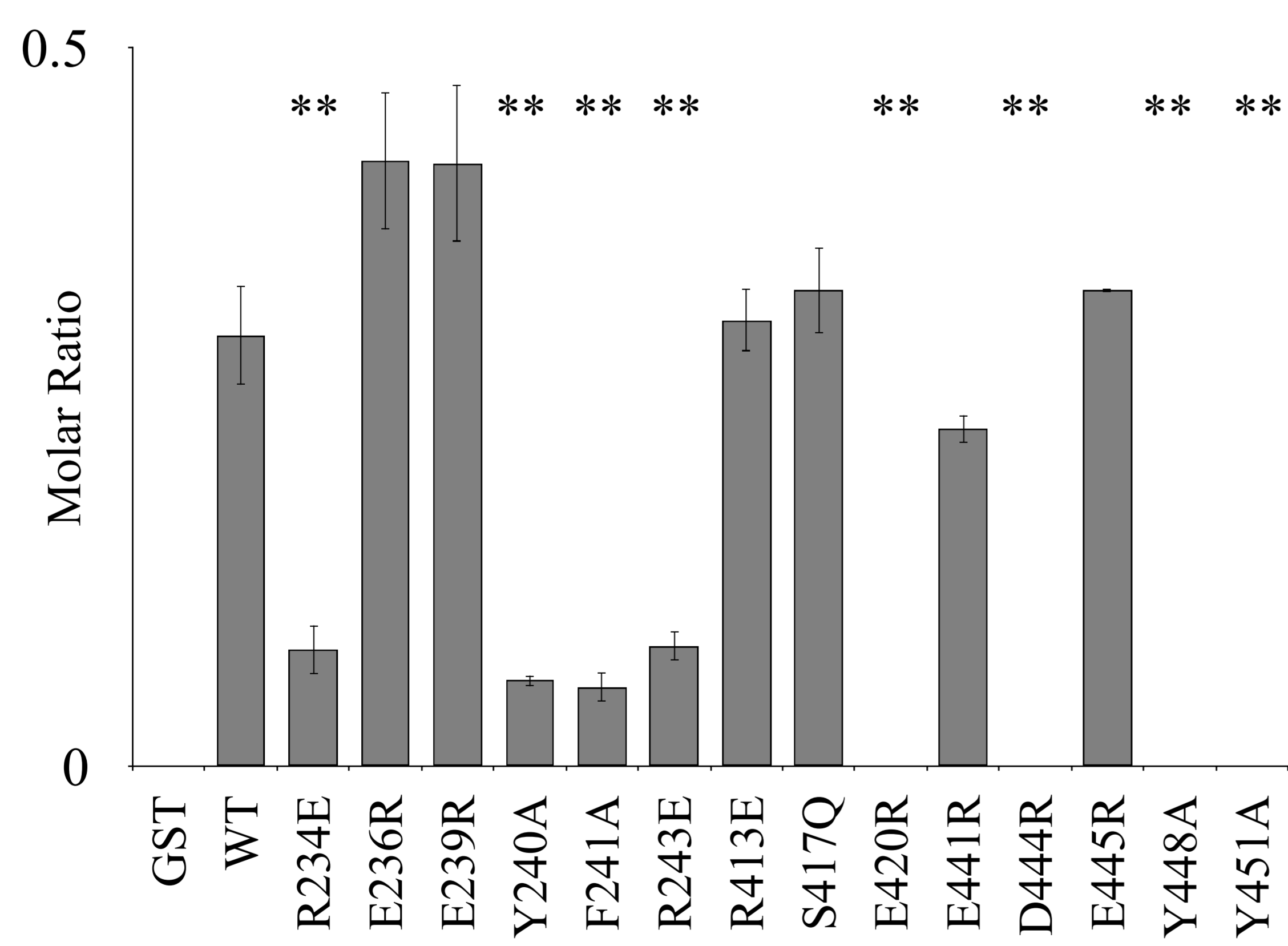**E**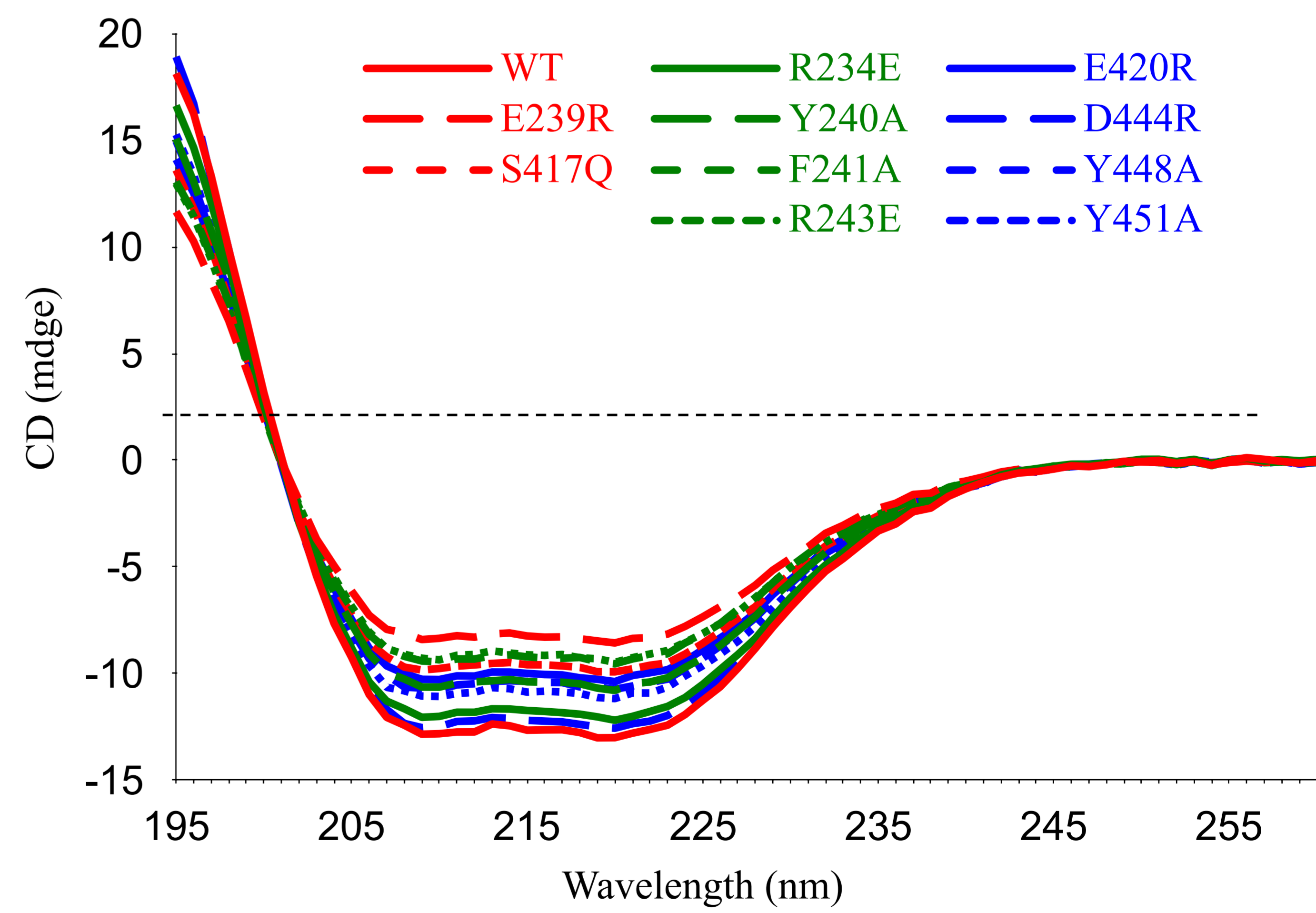



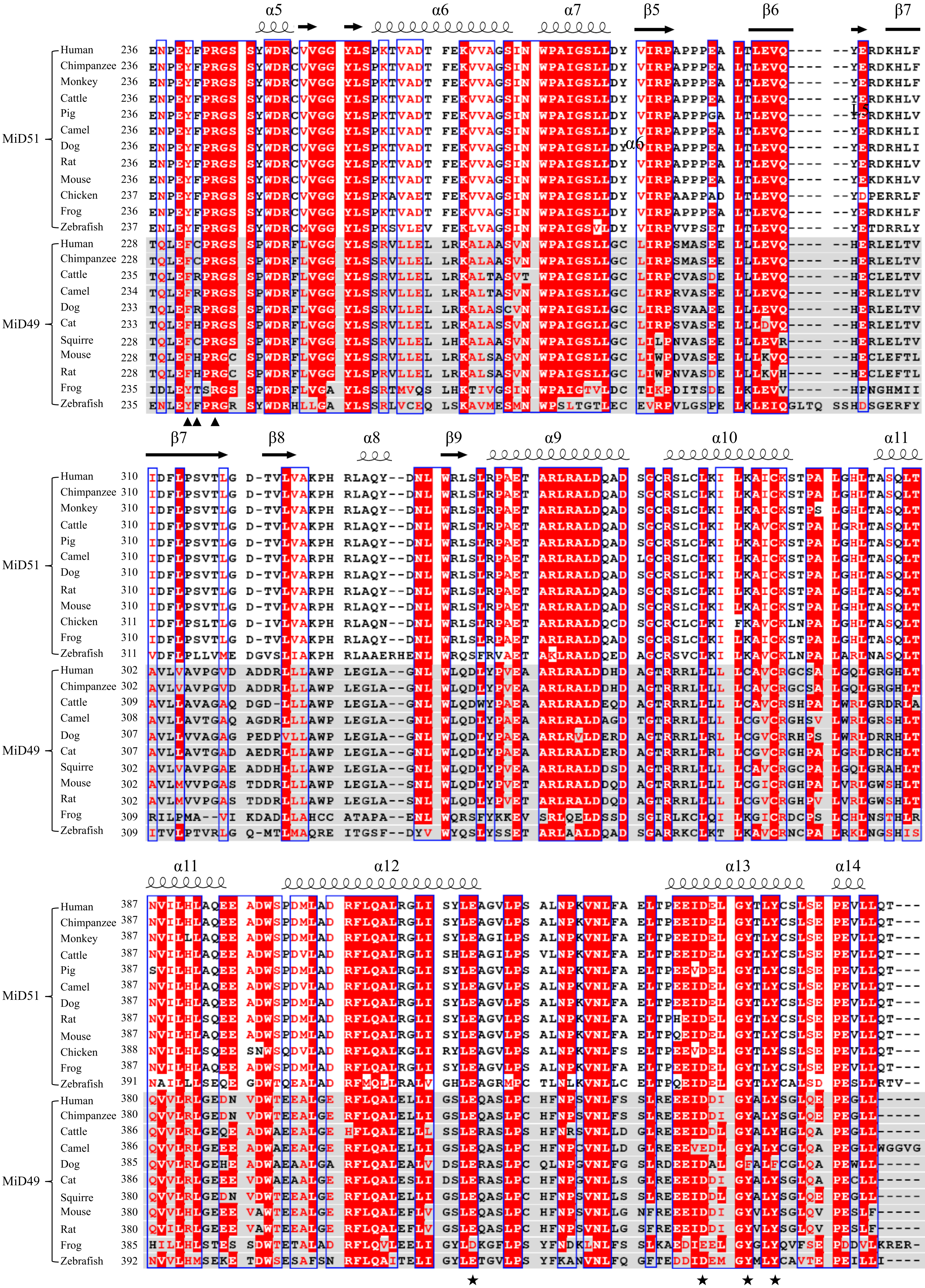

Supplement: S2 Fig — (A) Mutant forms of MiD51 containing clusters of three or four mutated residues were initially tested for ability to bind Drp1 with in vitro GST pull-down assays. Six MiD51 mutants that disrupt the interaction with Drp1 are colored in red. (B) Quantification of the results in (A). The binding affinity is expressed as molar ratio of Drp1 to MiD51 mutants. Data are shown as mean ± SEM of three independent experiments performed in triplicate, with ** P < 0.005 compared to wild-type. (C) In vitro GST pull-down assays were used to screen the single point mutants based on the results of (A) and (B). Mutations that disrupt the interaction with Drp1 are colored in red. (D) Quantification of the results in (C). The binding affinity is expressed as molar ratio of Drp1 to MiD51 mutants. Data are shown as mean ± SEM of three independent experiments performed in triplicate, with ** P < 0.005 compared to wild-type. (E) Circular dichroism spectroscopy confirmed that MiD51 mutants that have disrupted interactions with Drp1 still have the same conformation as wild type. (F) Sequence alignment of full-length MiD51 and MiD49 proteins. MiD51 and MiD49 proteins are distinguished by grey shading. Strictly conserved residues are highlighted in red, and moderately conserved residues are outlined in blue. Residues involved in Drp1 interaction are marked with ★ for DBS1 and ▲ for DBS2. The secondary structures are shown above the sequences. (PDF) [file pone.0211459.s002.pdf]

**A**

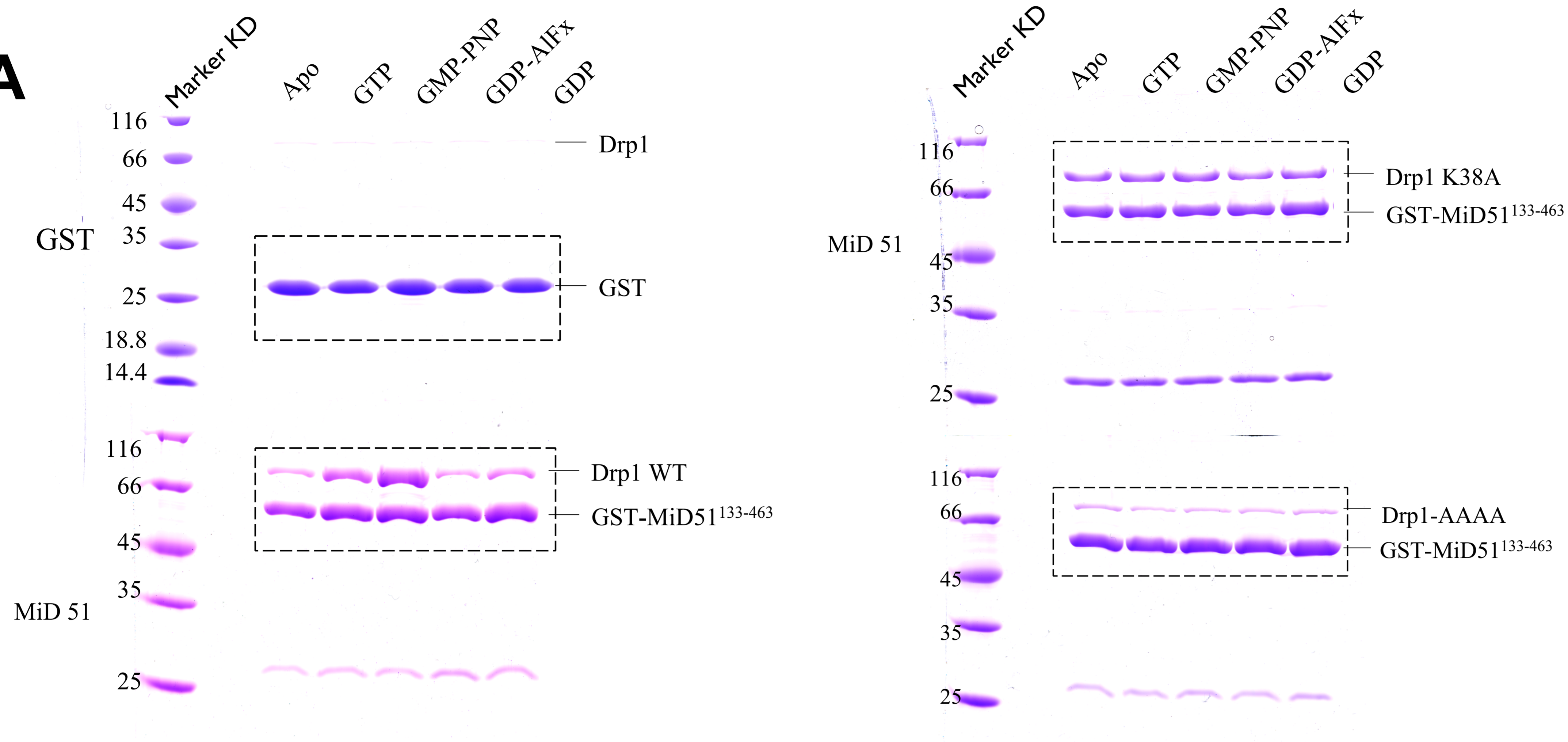

**B**

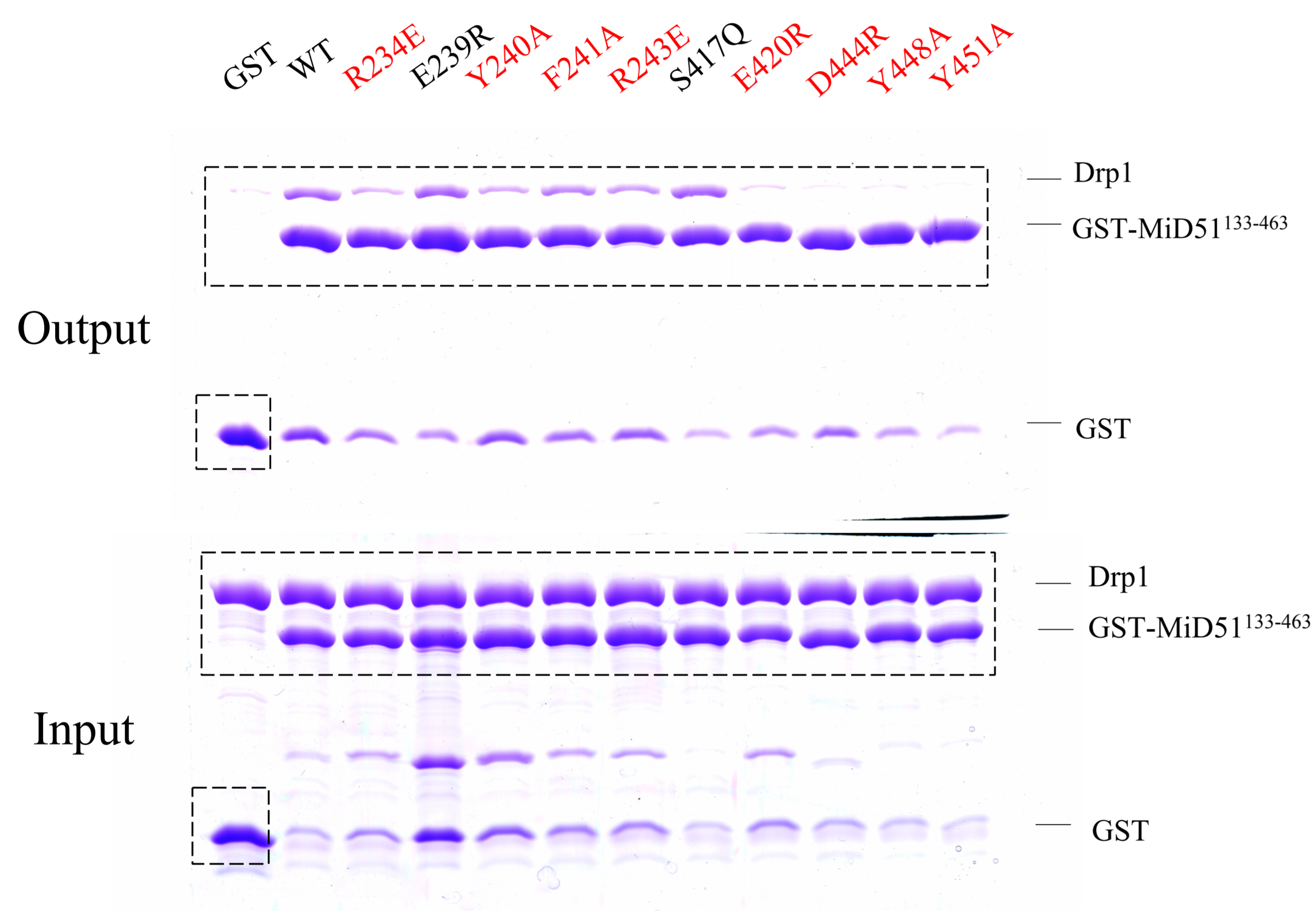

Supplement: S3 Fig — (A) Pull-down assays were performed to test the binding of purified Drp1 or mutants to GST-MiD51133-463 in the presence of different nucleotides, corresponding to Fig 1A. (B) WT and mutant GST-MiD51133-463 in vitro pull-down assays were performed with purified Drp1, corresponding to Fig 2C. (PDF) [file pone.0211459.s003.pdf]
