# Supplementary material for: New interfaces on MiD51 for Drp1 recruitment and regulation
Source: PLoS One. 2019 Jan 31;14(1):e0211459. doi: 10.1371/journal.pone.0211459 (PMC6355003; doi:10.1371/journal.pone.0211459)
Supplement: S1 Table — (DOCX) [file pone.0211459.s004.docx]

**S1 Table.** **Data collection and refinement statistics**

| Crystal | Se-MiD51^129-463^ | MiD51^133-463^ |
| --- | --- | --- |
| **Data collection** |  |  |
| Wavelength(Å) | 0.9793 | 0.9793 |
| Space group | P4_1_2_1_2 | P1 |
| Cell dimensions |  |  |
| *a*, *b*, *c* (Å) | 88.8, 88.8, 124.7 | 61.3, 64.7, 65.9 |
| α, β, γ (°) | 90.0, 90.0, 90.0 | 89.8, 108.1, 117.2 |
| Resolution (Å) | 50.00-2.70(2.80-2.70) | 50.00-1.85(1.92-1.85) |
| *R*_sym_ (%) | 0.087(0.832) | 0.068(0.595) |
| *I*/σ*I* | 15.9(2.1) | 23.9(5.4) |
| Completeness (%) | 99.6 | 95.3 |
| Redundancy | 4.4 | 5.5 |
| **Refinement** |  |  |
| Resolution (Å) | 44.42-2.70(2.80-2.70) | 48.54-1.85(1.92-1.85) |
| No. reflections | 14284 | 68988 |
| *R*_work_ / *R*_free_ (%) | 0.227/0.251 | 0.203/0.232 |
| **No. atoms** |  |  |
| Protein | 2592 | 5168 |
| Water | 10 | 202 |
| **B factors (Å2)** |  |  |
| Protein | 84.92 | 22.83 |
| Water | 71.01 | 27.94 |
| **r.m.s. deviations** |  |  |
| Bond lengths (Å) | 0.015 | 0.022 |
| Bond angles (°) | 2.08 | 2.16 |
