# Supplementary material for: New interfaces on MiD51 for Drp1 recruitment and regulation
Source: PLoS One. 2019 Jan 31;14(1):e0211459. doi: 10.1371/journal.pone.0211459 (PMC6355003; doi:10.1371/journal.pone.0211459)
Supplement: S2 Table — (DOC) [file pone.0211459.s005.doc]

**S2 Table. Sum of partial crystallographic statistics for MiD51^129-463^, MiD51^133-463^, and released PDB crystal structures**

| **PDB** | **Fragment** | **Crystal types** | **Space group** | **Unit-cell parameters** | **Molecules per asymmetric unit** | **Resolution** | **Reference** |
| --- | --- | --- | --- | --- | --- | --- | --- |
| 5X9B | 129-463 | Native | *P*4_1_2_1_2 | 88.8, 88.8, 124.7，  90.0, 90.0, 90.0 | 1 | 2.70 | This study |
| 5X9C | 133-463 | Native | *P*1 | 61.3, 64.7, 65.9  89.8, 108.1, 117.2 | 2 | 1.85 | This study |
| 4OAF | 134-463 | Native | *P*2_1_ | 91.1, 78.6, 102.3  90.0, 96.6, 90.0 | 4 | 2.20 | (Loson et al., 2014) |
| 4OAG |  | ADP bound | *P*2_1_ | 62.1, 80.8, 65.2  90.0, 105.7, 90.0 | 2 | 2.00 | (Loson et al., 2014) |
| 4OAH |  | H201A | *P*2_1_ | 82.4, 79.2, 103.5  90.0, 98.0, 90.0 | 4 | 2.00 | (Loson et al., 2014) |
| 4OAI |  | CDM | *P*2_1_2_1_2_1_ | 63.7, 67.1, 79.4  90.0, 90.0, 90.0 | 1 | 2.00 | (Loson et al., 2014) |
| 4NXT | 119-463 | Native | *P*1 | 72.7, 78.7, 79.4  66.3, 84.9, 64.1 | 4 | 2.12 | (Richter et al., 2014) |
| 4NXV |  | GDP bound | *P*1 | 72.3, 79.1, 80.1  65.8, 84.4, 64.1 | 4 | 2.30 | (Richter et al., 2014) |
| 4NXU |  | ADP bound | *P*1 | 72.6, 79.3, 79.4  65.4, 84.2, 63.4 | 4 | 2.30 | (Richter et al., 2014) |
| 4NXX |  | GDP bound | *P*4_3_2_1_2 | 57.7, 57.7, 255.4  90.0, 90.0, 90.0 | 1 | 2.55 | (Richter et al., 2014) |
| 4NXW |  | ADP bound | *P*4_3_2_1_2 | 57.7, 57.7, 253.8  90.0, 90.0, 90.0 | 1 | 2.55 | (Richter et al., 2014) |
