# Supplementary material for: New interfaces on MiD51 for Drp1 recruitment and regulation
Source: PLoS One. 2019 Jan 31;14(1):e0211459. doi: 10.1371/journal.pone.0211459 (PMC6355003; doi:10.1371/journal.pone.0211459)
Supplement: S3 Table — (DOC) [file pone.0211459.s006.doc]

**S3 Table. RMSD variations for superimposition of the C_α_backbone of MiD51^129-463^, MiD51^133-463^, and released PDB crystal structures**

| **RMSD** | | 1 | 2 | 3 | 4 | 5 | 6 | 7 | 8 | 9 | 10 |
| --- | --- | --- | --- | --- | --- | --- | --- | --- | --- | --- | --- |
|  |  | 4NXT | 4NXU | 4NXV | 4NXW | 4NXX | 4OAF | 4OAG | 4OAH | 4OAI | M129 |
| 2 | 4NXU | 1.13 |  |  |  |  |  |  |  |  |  |
| 3 | 4NXV | 0.91 | 0.38 |  |  |  |  |  |  |  |  |
| 4 | 4NXW | 1.40 | 1.13 | 1.14 |  |  |  |  |  |  |  |
| 5 | 4NXX | 1.40 | 1.07 | 1.18 | 0.18 |  |  |  |  |  |  |
| 6 | 4OAF | 0.54 | 1.19 | 0.98 | 1.45 | 1.45 |  |  |  |  |  |
| 7 | 4OAG | 0.94 | 1.09 | 0.95 | 1.57 | 1.48 | 0.88 |  |  |  |  |
| 8 | 4OAH | 1.09 | 0.76 | 0.75 | 1.44 | 1.46 | 1.07 | 0.71 |  |  |  |
| 9 | 4OAI | 1.39 | 2.09 | 1.90 | 1.92 | 1.89 | 1.37 | 1.54 | 1.87 |  |  |
| 10 | M129 | 1.66 | 1.86 | 1.78 | 1.79 | 1.80 | 1.64 | 1.85 | 1.47 | 1.65 |  |
| 11 | M133 | 1.47 | 1.73 | 1.62 | 1.92 | 1.88 | 1.44 | 1.65 | 1.22 | 0.97 | 1.14 |
