# Supplementary material for: New interfaces on MiD51 for Drp1 recruitment and regulation
Source: PLoS One. 2019 Jan 31;14(1):e0211459. doi: 10.1371/journal.pone.0211459 (PMC6355003; doi:10.1371/journal.pone.0211459)
Supplement: S4 Table — (DOC) [file pone.0211459.s007.doc]

**S4 Table.** **Mutation screening of residues on MiD51 interacting with Drp1**

| **Mutations** | **Interactions** | **Mutations** | **Interactions** |
| --- | --- | --- | --- |
| WT | / | D402R/D406R/Q410T | Maintained |
| S134L/E137R/K138E/T141Q | Maintained | R413E/S417Q/E420R | Abolished |
| 144-RNR-146→ELE | Maintained | E441R/D444R/E445R | Abolished |
| R156E/Q159T/D163R | Maintained | Y448A/Y451A | Abolished |
| E167R/S170Q/R173E | Maintained | C452S | Maintained |
| 177-PDMP-180→AAA | Maintained | E456R | Maintained |
| 182-RDMY-185→AAA | Maintained | E458R | Maintained |
| 191-YDDLQ-195→AAA | Maintained | V459E | Maintained |
| 196-VVTADH-201→AAA | Maintained | Q462F | Maintained |
| 211-EQN-213→AAA | Maintained | T463D | Maintained |
| 221-EDTIMN-226→AAA | Maintained | R234E | Weakened |
| 234-RREN-237→AAA | Weakened | E236R | Maintained |
| Δ(238-243) | Weakened | E239R | Maintained |
| Δ(234 -243) | Weakened | Y240A | Weakened |
| Y256A | Maintained | F241A | Weakened |
| K260E/D264R/K268E | Maintained | R243E | Weakened |
| 291-PPPE-294→AAA | Maintained | R413E | Maintained |
| 303-ERDK-306→AAAA | Maintained | S417Q | Maintained |
| D320R | Maintained | E420R | Abolished |
| 326-KPH-328→AAA | Maintained | E441R | Maintained |
| 329-RLAQYDN-335→AAAA | Maintained | D444R | Abolished |
| R348E/R350E/Q354T | Maintained | Y445R | Maintained |
| K372R | Maintained | Y448A | Abolished |
| 394-QEE-396→TRR | Maintained | Y451A | Abolished |
